# SSX expression and function in prostate cancer

**DOI:** 10.1186/2051-1426-1-S1-P47

**Published:** 2013-11-07

**Authors:** Jordan E Bloom, Douglas G McNeel

**Affiliations:** 1 University of Wisconsin-Madison, Madison, WI, USA

## Background

Cancer-testis antigens (CTAs) are a group of proteins whose expression is essentially restricted to testis germline cells among normal somatic cells. CTAs also possess a heightened expression in many different types of cancer. Due to their restricted expression in tumor cells among MHC class I-expressing cells, there has been interest in the CTA as essentially tumor-specific targets for CD8+ T cell-directed therapies. Amongst the CTA genes is the Synovial Sarcoma X chromosome breakpoint (SSX) gene family. There are ten members of the SSX family with a high degree of homology amongst them. SSX-2 is the most commonly expressed member of the SSX family, and serves as a prototypical member for study. We have previously demonstrated that SSX-2 expression is confined to metastatic tissue, and is not detected in primary prostate tumors. The function of the SSX family is largely unknown, and further insights into its function may reveal avenues for future directed therapies.

## Methods

We determined the pattern of SSX-2 expression in patient peripheral blood; RT-PCR was used on peripheral blood samples from patients with prostate cancer as well as healthy controls. To determine the identity and prevalence of SSX family members in metastatic tissue, cDNA from metastatic tissue was examined by PCR with primers specific for each SSX family member. To further understand SSX-2’s function, SSX-2 expression was knocked down in the 22Rv1 cell line using an siRNA plasmid. This cell line was then assayed for tumorigenicity (soft agar colony formation), proliferation, and invasion (wound healing assay). Finally using qPCR, expression levels of markers of the epithelial to mesenchymal transition (EMT) were examined in the knocked down 22Rv1 cell line.

## Results

We have detected SSX-2 mRNA in the peripheral blood of 17 of 33 (51%) patients with prostate cancer with circulating prostate tumor cells and 0% (0 of 5) control blood donors without prostate cancer. SSX-2 was found to be in 47% (7/15) of metastatic cDNA samples, SSX-1 was found at a lower rate (1/15), and no additional family members were detected. In an SSX-2 knockdown of the 22Rv1 cell line, EMT markers Slug and Vimentin were shown to have their expression modulated. Additionally, the 22Rv1 SSX-2 knockdown line has demonstrated a higher capacity to form colonies in soft agar.

## Conclusions

Due to SSX-2’s is preferential expression, as well as the effect of SSX knockdown on known EMT markers and soft agar colony formation, we believe that SSX-2 is involved in the EMT process, and potentially in the progression of prostate cancer to metastatic disease.

